# Assessment of intra-tumoural colorectal cancer prognostic biomarkers using RNA *in situ* hybridisation

**DOI:** 10.18632/oncotarget.26675

**Published:** 2019-02-15

**Authors:** Arthur Morley-Bunker, John Pearson, Margaret J. Currie, Helen Morrin, Martin R. Whitehead, Tim Eglinton, Logan C. Walker

**Affiliations:** ^1^ Mackenzie Cancer Research Group, Department of Pathology and Biomedical Science, University of Otago, Christchurch, New Zealand; ^2^ Biostatistics and Computational Biology Unit, University of Otago, Christchurch, New Zealand; ^3^ Cancer Society Tissue Bank, Department of Pathology and Biomedical Science, University of Otago, Christchurch, New Zealand; ^4^ Canterbury Health Laboratories, Christchurch Hospital, Christchurch, New Zealand; ^5^ Department of Surgery, University of Otago, Christchurch, New Zealand

**Keywords:** colorectal cancer, gene expression, RNA in situ hybridisation, prognostic markers

## Abstract

Genome-wide expression studies using microarrays and RNAseq have increased our understanding of colorectal cancer development. Translating potential gene biomarkers from these studies for clinical utility has typically relied on PCR-based technology and immunohistochemistry. Results from these techniques are limited by tumour sample heterogeneity and the lack of correlation between mRNA transcript abundance and corresponding protein levels. The aim of this research was to investigate the clinical utility of the RNA *in situ* hybridisation technique, RNAscope^®^, for measuring intra-tumoural gene expression of potential prognostic markers in a colorectal cancer cohort. Two candidate gene markers (*GFI1* and *TNFRSF11A*) assessed in this study were identified from a previous study led by the The Cancer Genome Atlas (TCGA) Network, and analysis was performed on 112 consecutively collected, archival FFPE colorectal cancer tumour samples. Consistent with the TCGA Network study, we found reduced *GFI1* expression was associated with high-grade and left-sided tumours, and reduced *TNFRSF11A* expression was associated with metastasis and high nodal involvement. RNAscope^®^ combined with image analysis also enabled quantification of *GFI1* and *TNFRSF11A* mRNA expression levels at the single cell level, allowing cell-type determination. These data showed that reduced mRNA transcript abundance measured in patients with poorer prognosis occurred in carcinoma cells, and not lymphocytes, stromal cells or normal epithelial cells. To our knowledge, this is the first study to assess the intra-tumoural expression patterns of *GFI1* and *TNFRSF11A* and to validate their microarray expression profiles using RNAscope. We also demonstrate the utility of RNAscope^®^ technology to show that expression differences are derived from carcinoma cells rather than from cells located in the tumour microenvironment.

## INTRODUCTION

The diagnosis of colorectal cancer occurs primarily by histological examination of tissue specimens obtained during an endoscopic procedure, or at the time of surgical resection [1]. Accurate staging is essential, as it used to predict disease prognosis and influence treatment options for individual patients. However, current staging is not completely accurate as evidenced by the fact that up to 25% of stage II patients will suffer recurrence [2, 3]. Given these shortcomings in existing clinicopathological staging, interest has focused on molecular biomarkers that have the potential to predict prognosis and guide therapy more accurately for individual patients [4]. While a number of biomarkers have been proposed, few have been implemented in routine clinical practice.

Our understanding of the molecular basis of colorectal cancer has increased with the advent of molecular technologies that enable genome-wide expression analysis, such as microarrays and massively parallel sequencing [5, 6]. In 2012, The Cancer Genome Atlas Network (TCGA) reported a comprehensive study of 276 colorectal tumour samples, which utilised sequencing technologies to analyse tumour exome sequence, DNA copy number, promoter methylation, and expression of messenger RNA and microRNA [7].The study provided key insights into colorectal cancer biology, including identification of potential prognostic and therapeutic targets. However, as with other genomic profiling studies, there has been a paucity of candidate markers translated into clinical practice [4, 8].

The inconsistencies seen may be due to the confounding effect of tumour heterogeneity combined with limitations in PCR-based technology and immunohistochemistry. The quantitative PCR-based method might be considered the ‘gold standard’ for measuring gene expression because of its high sensitivity and specificity. However, the sensitivity of this method is limited if applied to heterogeneous multicellular tumour samples, and it is prone to interference from non-cancerous cells. Immunohistochemistry has proven to be an integral method for measuring protein expression levels in tissue samples for diagnostic laboratories, however it is less quantitative than other molecular methods [9]. Furthermore, many genes show a low correlation between mRNA transcript expression and corresponding protein levels [10], and only 25% of the proteins within the human proteome have validated antibodies [11]. The limitations that tumour heterogeneity imposes upon PCR-based methods and IHC can be resolved to an extent with the use of laser microdissection to select cells of interest to produce an enriched population of cells for analysis [12–14]. However, this requires further investment in additional hardware; time and reliance on pathologist/scientist to correctly identify target cells of interest. More suitable methods are therefore required to verify and assess the application of potential diagnostic and prognostic markers whilst taking into account the effects of tumour heterogeneity. RNAscope^®^ (Advanced Cell Diagnostics, Inc. (Hayward, CA) is one such method that can satisfy these requirements.

RNAscope^®^ is an RNA *in situ* hybridisation method that enables single-molecule detection while preserving cellular and tissue morphology [15]. This method utilises a paired probe system to ensure sensitive and selective targeting of mRNA molecules [16], and has been successfully applied to measure gene expression in cancer cells [17–19]; stem cells [20–22] and circulating tumour cells [23, 24]. RNAscope^®^ allows mRNA expression measurements for individual or multiple genes from archival tumour specimens to be obtained in a diagnostic setting [15]. Furthermore, a strong correlation of mRNA expression has been shown between RNAscope^®^ and quantitative PCR [25].

The purpose of this study was to investigate the utility of RNAscope^®^ for measuring mRNA expression levels of genes previously associated with poor prognostic outcomes in colorectal cancer, and determine mRNA transcript localisation and expression in different cell types within the tumour.

## RESULTS

### Comparison between copy number and mRNA expression level

**Figure 1:**
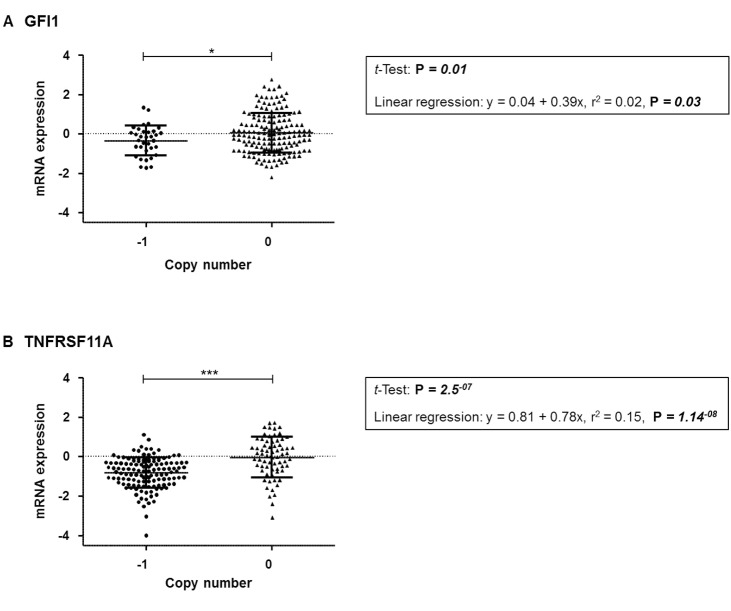
Comparison of mRNA expression relative to copy number from combined TCGA datasets for the respective gene (**A**) *GFI1* (**B**) *TNFRSF11A*. Significant *p*-values are: < 0.001 = ^***^, < 0.05 = ^*^.

To confirm the possibility that mean mRNA expression levels for *TNFRSF11A* and/or *GFI1* can be affected by CNA, analysis of mRNA expression levels and CNA was performed using TCGA data. From a total of 629 patients, 218 had DNA copy number and gene expression data. *GFI1* and *TNFRSF11A*, showed a significant association between predicted gene CNA and expression levels for both *GFI1* (average difference between loss and neutral was −0.39, CI −0.70 − −0.08, *P* = 0.01) and *TNFRSF11A* (average difference between loss and neutral was −0.78, CI −1.06 – −0.50, *P* = 2.5E^−07^) with a trend of CNA deletions being associated with mRNA downregulation (Figure 1).

### Comparison of RNA *in situ* hybridization and immunohistochemistry assessment

**Figure 2:**
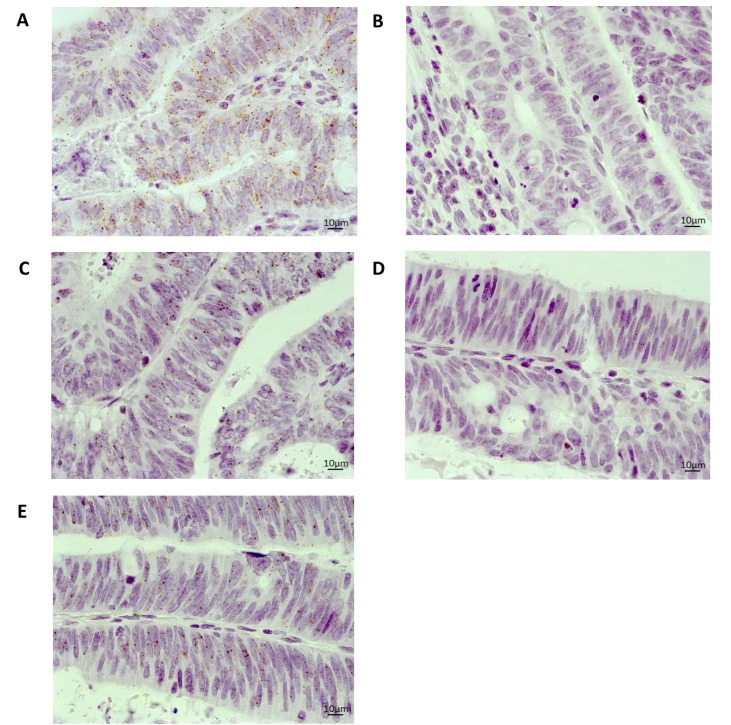
Representative images of mRNA expression using RNAscope^®^ on an FFPE whole tissue section from a colorectal cancer case 40× magnification (**A**) Positive control (*PPIB*) (**B**) Negative control (DAB only) (**C**) *MLH1* (**D**) *GFI1* (**E**) *TNFRSF11A*.

Expression of *MLH1* mRNA has been correlated closely with protein expression [20, 21], so this gene was selected to compare the utility of RNAscope^®^ with a routinely used immunohistochemical diagnostic assay. RNAscope^®^ and immunohistochemistry assays were performed on a total of 112 cases. Microscopic assessment of *MLH1* mRNA expression showed clean staining (brown punctuate dots) with no background staining or staining artefacts (Figure 2). Interestingly, different mRNA expression patterns were observed in cases. A homogenous mRNA expression pattern where *MLH1* probe signals were evenly dispersed throughout the tumour was found in 8 cases. These cases were more readily identifiable and generally scored higher (i.e. had more signals per cell) than 43 cases that presented a heterogeneous expression with *MLH1* probe signals observed in localised areas of the tumour.

Immunohistochemistry was successfully performed with the majority of cases demonstrating positive staining of MLH1 within carcinoma cells. However, staining intensities did vary between cases.

Comparison of results from these technologies correlated in 55.0% (62/112) of cases (Table 1). Positive mRNA expression correlated with positive protein expression in 57.0% (53/93) of cases. A negative mRNA expression correlated with a negative protein expression in 64.3% (9/14) of cases. Re-analysis of cases with good quality RNA present resulted in stronger concordance between RNAscope^®^ and immunohistochemistry in 73.7% (56/76) of cases whilst 29 cases were excluded (Table 2). RNAscope^®^ and immunohistochemistry were found not to be independent of each other (OR = 6.0; CI 1.59−22.62, *P* = 0.007, Fisher's Exact test). A significant positive correlation between mRNA and protein expression was observed in 75.0% (48/64) of cases (*P* = 0.018). A negative mRNA expression correlated with a negative protein expression in 66.6% (8/12) of cases. Investigation of the discrepant cases found 16 cases with positive MLH1 protein expression had no detectable *MLH1* mRNA expression present. Conversely, four cases with negative MLH1 protein expression, showed positive *MLH1* mRNA expression. Such differences highlight potential variations in half-life between molecules and/or sensitivities between technologies. We were not able to evaluate these findings in relation to *MLH1* mutation or methylation status, however, the results further demonstrate the utility of RNAscope^®^ for measuring gene mRNA expression changes in archival FFPE tissue.

**Table 1:** Assessment of *MLH1* mRNA expression using RNAscope^®^ and *MLH1* protein expression using immunohistochemistry

		Immunohistochemistry			
		Negative	Positive	Loss of tissue	Total
**RNAscope^®^**	Negative	9	39	0	48
	Positive	4	53	0	57
	No Result Available	1	1	5	7
	Total	14	93	5	112

**Table 2:** *MLH1* mRNA expression using RNAscope^®^ and *MLH1* protein expression using immunohistochemistry from samples with a positive control score of 2+

		Immunohistochemistry			
		Negative	Positive	Total
**RNAscope^®^**	Negative	8	16	24
	Positive	4	48	52
	Total	12	64	76

### RNA *in situ* hybridisation assessment of *GFI1* and *TNFRSF11A* expression in colorectal cancer patients

We observed both homogenous and heterogeneous *GFI1* and *TNFRSF11A* mRNA expression patterns across the tumour cohort. For *GFI1*, there were 3 cases with homogenous mRNA patterns and 9 cases with heterogeneous expression. For *TNFRSF11A*, a homogenous expression pattern was observed in 11 cases, whilst 28 cases showed a heterogeneous expression pattern. Colorectal adenocarcinoma cases were analysed for associations between *GFI1* or *TNFRSF11A* mRNA expression and clinicopathological features. Significant associations between negative *GFI1* mRNA expression and age groups (CI 0.55–12.27, *P* = 0.05), grade (CI 0.003–0.91, *P* = 0.05), left-sided tumours (CI 1.21–21.45, *P* = 0.02), and rectal tumours (CI 0.07 – 2.26, *P* = 0.04) were observed by univariate analysis (Table 3).

**Table 3:** *GFI1* mRNA expression and association with clinicopathological features from colorectal cancer adenocarcinoma cases from the RNAscope cohort that had an RNAscope^®^ positive control score of > 2

		RNAscope^®^				
Clinicopathological features		−ve	+ve	OR	95% CI	*P*^a^
**Age**	30–65	14	3	1		***0.05***
	66–75	26	2	0.36	[0.04, 2.41]	
	76+	16	8	2.33	[0.55, 12.27]	
**Gender**	Female	24	4	1		0.42
	Male	32	9	1.69	[0.49, 6.83]	
**Grade**	1-Well differentiated	1	3	1		***0.05***
	2-Moderately differentiated	43	8	0.06	[0.01, 0.67]	
	3-Poorly differentiated	11	2	0.06	[0.003, 0.91]	
**Grade Status**	Low	45	11	1	-	0.72
	High	11	2	0.74	[0.30, 9.50]	
**Lymphatic Invasion**	Absent	38	9	1		0.93
	Present	18	4	0.94	[0.81, 1.21]	
**Vascular Invasion**	Absent	42	11	1		0.47
	Present	14	2	0.55	[0.74, 1.15]	
**Lymphovascular Invasion**	Absent	38	9	1		0.92
	Present	18	4	0.94	[0.23, 3.31]	
**Metastasis Present**	Absent	37	9	1		0.83
	Present	19	4	0.87	[0.21, 3.04]	
**Site**	Left-sided	32	3	1		***0.02***
	Right-sided	24	10	4.4	[1.21, 21.45]	
**Primary Site**	Ascending/Caecum	24	10	1	-	***0.04***
	Descending/Sigmoid	22	1	0.10	[0.01, 0.63]	
	Rectum	10	2	0.48	[0.07, 2.26]	
**AJCC Pathologic Tumour Stage**	T1	16	6	1		0.23
	T4	40	7	0.47	[0.13, 1.65]	
**AJCC Nodal Stage**	N0	36	9	1	-	0.73
	N1+N2	20	4	0.8	[0.20, 2.80]	
**AJCC Metastatic Stage**	M0 -unconfirmed	53	13	1		0.26
	M1 -confirmed	3	0	0	[0.00, NaN]	
**AJCC Tumour Stage Group**	1	11	5	1	-	0.37
	2	25	4	0.35	[0.07, 1.57]	
	3 + 4	20	4	0.44	[0.09, 1.99]	
**Survival**	Alive	44	10	1	-	0.90
	Deceased	12	3	1.10	[0.22, 4.30]	

^a^RNAscope data has univariate *P* value from Logistics’ regression.

Abbreviations: AJCC, American Joint Committee on Cancer; CI, Confidence Interval.

Analysis of *TNFRSF11A* mRNA expression and clinicopathological features showed significant associations for negative *TNFRSF11A* mRNA expression and presence of metastasis (CI 0.13 – 1.04, *P* = 0.05), and AJCC Nodal Stage involvement (CI 0.11 – 0.92, *P* = 0.03) (Table 4). No associations were observed between expression of *GFI1* and/or *TNFRSF11A* with the clinicopathological features, histology, tumour size, lymphocytic infiltrate. No information relating to histological type (mucinous versus non-mucinous) was available for this study.

**Table 4:** *TNFRSF11A* mRNA expression and association with clinicopathological features form colorectal adenocarcinoma cases from the RNAscope cohort that had an RNAscope^®^ positive control score of > 2

		RNAscope^®^				
Clinicopathological Features		−ve	+ve	OR	95% CI	*P*^a^
**Age**	30–65	9	8	1	-	0.65
	66–75	15	13	0.98	[0.29, 3.31]	
	76+	10	14	1.58	[0.45, 5.64]	
**Gender**	Female	10	18	1	-	0.06
	Male	24	17	0.39	[0.14, 1.04]	
**Grade**	1-Well differentiated	1	3	1	-	0.07
	2-Moderately differentiated	24	27	0.38	[0.04, 3.85]	
	3-Poorly differentiated	8	5	0.21	[0.02, 2.60]	
**Grade Status**	Low	26	30	1	-	0.32
	High	8	5	0.54	[0.16, 1.86]	
**Lymphatic Invasion**	Absent	21	26	1	-	0.27
	Present	13	9	0.56	[0.67, 1.12]	
**Vascular Invasion**	Absent	23	30	1		0.07
	Present	11	5	0.35	[0.59, 1.02]	
**Lymphovascular Invasion**	Absent	21	26	1	-	0.26
	Present	13	9	0.56	[0.20, 1.55]	
**Metastasis Present**	Absent	19	27	1	-	***0.05***
	Present	15	8	0.38	[0.13, 1.04]	
**Site**	Left-sided	17	18	1		0.91
	Right-sided	17	17	0.94	[0.37, 2.44]	
**Primary Site**	Ascending/Caecum	17	17	1	-	0.70
	Descending/Sigmoid	10	13	1.30	[0.45, 3.83]	
	Rectum	7	5	0.71	[0.18, 2.68]	
**AJCC Pathologic Tumour Stage**	T1	8	14	1		0.14
	T4	26	21	0.46	[0.16, 1.29]	
**AJCC Nodal Stage**	N0	18	27	1	-	***0.03***
	N1 + N2	16	8	0.33	[0.11, 0.92]	
**AJCC Metastatic Stage**	M0 -unconfirmed	33	33	1		0.57
	M1 -confirmed	1	2	2	[0.18, 44.27]	
**AJCC Tumour Stage Group**	1	5	11	1	-	0.07
	2	13	16	0.56	[0.14, 1.97]	
	3 + 4	16	8	0.23	[0.05, 0.84]	
**Survival**	Alive	25	29	1	-	0.35
	Deceased	9	6	0.57	[0.17, 1.82]	

^a^RNAscope data has univariate *P* value from Logistic Regression.

Abbreviations: AJCC, American Joint Committee on Cancer; CI, Confidence Interval.

### Quantitative assessment of *GFI1* and *TNFRSF11A* mRNA expression in different tumour cell types

**Figure 3:**
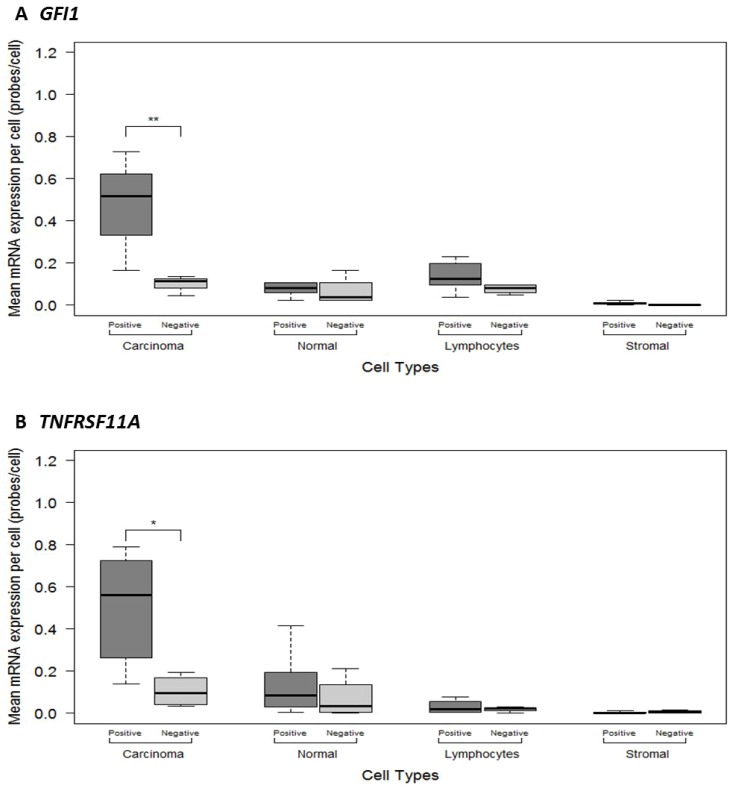
ImageJ manual method quantitative measurements of mRNA expression levels in different cells types between the 10 colorectal cancer FFPE histological sections categorised as showing Negative or Positive mRNA expression for (**A**) *GFI1* (**B**) *TNFRSF11A*. Significant *p*-values are: <0.001 = ^***^, <0.01 = ^**^, <0.05 = ^*^.

To better characterise the intra-tumoural expression patterns of *GFI1* and *TNFRSF11A,* we used RNAscope^®^ to quantify mRNA levels across 10 whole tumour sections according to tumour cell type (Figure 2 and Supplementary Figure 1). This analysis showed that mean *GFI1* and *TNFRSF11A* mRNA expression levels within carcinoma cells were significantly higher compared to other cell types (*F* = 14.86, *P* = 1.89E-08 and *F* = 10.27, *P* = 1.11E-06), including normal epithelial cells, stromal fibroblasts and lymphocytes (Figure 3 and Table 5). The mean expression levels for *GFI1* in carcinoma cells was 4.7-fold higher than surrounding cell types (0.33 vs 0.07 signals/cell; CI 0.08 – 0.44, *P* = 0.009). The mean expression levels for *TNFRSF11A* in carcinoma cells was 7-fold higher than surrounding cell types (0.35 vs 0.05 signals/cell; CI 0.09 – 0.51, *P* = 0.009). No statistical significance was observed for differences in mean mRNA expression when comparing amongst other cell types.

**Table 5:** Comparison of the mean *GFI1* and *TNFRSF11A* mRNA expression levels on a per cell basis between different cell types

Gene	Cell type	mean mRNA signals/cell	95% CI	*p*-value	ANOVA *F*-statistic	ANOVA *p*-value
**GFI1**	Carcinoma vs All Cell Types	0.33 vs 0.07	0.08, 0.44	***0.009***	***14.86***	***1.89E-08***
	Carcinoma vs Normal	0.33 vs 0.09	0.08, 0.41	***0.001***		
	Carcinoma vs Lymphocytes	0.33 vs 0.11	0.06, 0.38	***0.004***		
	Carcinoma vs Stromal	0.33 vs 0.01	0.16, 0.49	***2.25E-05***		
	Normal vs Lymphocytes	0.09 vs 0.11	−0.19, 0.14	0.971		
	Normal vs Stromal	0.09 vs 0.01	−0.24, 0.08	0.538		
	Stromal vs Lymphocytes	0.01 vs 0.11	−0.27, 0.05	0.296		
**TNFRSF11A**	Carcinoma vs All Cell Types	0.35 vs 0.05	0.09,0.51	***0.009***	***10.27***	***1.11E-06***
	Carcinoma vs Normal	0.35 vs 0.11	0.05, 0.43	***0.001***		
	Carcinoma vs Lymphocytes	0.35 vs 0.03	0.13, 0.32	***3.63E-04***		
	Carcinoma vs Stromal	0.35 vs 0.01	0.15, 0.34	***1.49E-04***		
	Normal vs Lymphocytes	0.11 vs 0.03	−0.11, 0.08	0.641		
	Normal vs Stromal	0.11 vs 0.01	−0.30, −0.11	0.459		
	Stromal vs Lymphocytes	0.01 vs 0.03	−0.21, −0.02	0.991		

Abbreviations: CI, (Confidence Intervals); HSD (honest significant difference). Carcinoma tested against All cell types with a 2 sample *t*-test, all other tests were Tukey's HSD tests.

## DISCUSSION

Candidate markers from large expression profiling studies have made little clinical impact to date, possibly due to the confounding effect of heterogeneous tumour samples. RNA *in situ* hybridisation can overcome these limitations by measuring mRNA expression at the single cell level.

This study successfully established RNA *in situ* hybridisation (RNAscope^®^) as a method for assessing mRNA expression on archival FFPE material in a similar manner to immunohistochemistry for assessing protein expression. This study then utilized RNAscope^®^ to quantify mRNA expression of candidate prognostic markers *GFI1* and *TNFRSF11A,* and analysed their association with clinicopathological features from FFPE colorectal tumour tissue. Furthermore, this study quantified intra-tumoural expression of *GFI* and *TNFRSF11A* in different cell types present within the colorectal tumour tissue.

MLH1 immunohistochemistry is used, in conjunction with other genes, as a diagnostic assay to ascertain whether a colorectal cancer patient has a deficient mismatch repair system (microsatellite instability) [4]. Comparing RNAscope^®^ data with immunohistochemistry showed a correlation between RNA and protein levels for *MLH1* demonstrating the utility of RNAscope^®^ against an established diagnostic tool. However, despite the use of a positive control gene to ensure RNA integrity in each core, some discrepancies between detection of mRNA and protein expression were encountered. Possible explanations include variable immunohistochemical staining intensities being misinterpreted; the likelihood that not all *MLH1* mRNA is translated into protein due to post-translational modifications, or that protein degradation is occurring. Additionally, tumour stage-related changes may also affect mRNA expression levels during tumour progression [7]. Interestingly, previous studies did not find strong correlation between MLH1 mRNA and protein expression in colorectal tumours either [26, 27].

Having established RNAscope^®^ as a technical comparable method to immunohistochemistry, this study successfully utilized RNA *in situ* hybridisation (RNAscope^®^) to quantify mRNA expression of candidate prognostic markers *GFI1* and *TNFRSF11A* from FFPE colorectal tumour tissue. Data from RNAscope^®^ showed associations for reduced *GFI1* and *TNFRSF11A* mRNA expression to poor prognostic features, similar to those identified using microarray expression data from a large TCGA study [7].

Reduced expression of *GFI1* was significantly associated with left sided and rectal (primary site) tumours. This is in contrast to the observation that left-sided colorectal cases have a better prognosis than right-sided tumours, even when adjusting for possible differences in screening practices and treatment [28]. Our results could be identifying a subset of distal tumours that have poor prognostic outcomes, reflecting differences in tumour biology. This would help contribute to the understanding of molecular subtypes of colorectal cancer and possible prognostic implications. Further studies are required to confirm these findings.

Our RNAscope^®^ data also showed good agreement with the TCGA study [7] for reduced expression of *TNFRSF11A* and tumour features consistent with poor prognosis, including high nodal stage and metastatic disease. The role of *TNFRSF11A* in colorectal cancer tumorigenesis remains to be elucidated. There are limited studies reporting *TNFRSF11A* mRNA expression in colorectal cancer cases [29]. Santini *et al*., reported TNFRSF11A overexpression in primary tumours of colorectal cancer cases and their associated metastases to the bone [29]. In contrast, our results and those of the previous TCGA study [7], show reduced *TNFRSF11A* mRNA expression is associated with poor prognosis. A possible explanation is that reduced TNFRSF11A mRNA expression alters the *TNFRSF11A /TNFSF11/TNFRSF11B* signalling axis in cells. Reduced *TNFRSF11A* expression leads to reduced availability of TNFSF11, promoting more TNFRSF11B to be available. TNFRSF11B acts as a decoy receptor for TRAIL resulting in decreased TRAIL induced apoptosis, thus allowing cells to survive for longer and proliferate [30]. Furthermore, cells are able to secrete cytokines into the tumour microenvironment to promote an inflammatory state to recruit tumour associated macrophages that support continued tumour cell growth and progression [31]. Alternatively, reduced *TNFRSF11A* mRNA expression may be associated with a specific molecular subtype(s) of colorectal cancer. A limitation with this study is that molecular subtyping information of cases was not available. Further work is required to determine the role of *TNFRSF11A* in colorectal tumorigenesis.

Unlike the TCGA study [7], we did not observe an association between *GFI1* and lymphatic invasion, fraction of positive lymph nodes (nodal involvement), tumour stage and distant metastasis. Additionally, we did not observe an association between *TNFRSF11A* and tumour stage or distant metastasis. These differences may be due to the level of power associated with the smaller cohort used in this study. This limitation affected our ability to perform multivariate analysis due to the small proportion of cases within certain categories.

Differences between RNAscope^®^ and the TCGA study [7] may also reflect the selection criteria for the genes used in this study. *GFI1* was selected for its high statistical significance ranking for mRNA expression levels and association with prognostic features. Similarly, *TNFRSF11A* was selected for its high statistical significance ranking of mRNA expression and importantly for the first gene to be known to be affected by CNA status. Analysis of the data downloaded from the cBioPortal demonstrated a statistically significant association between negative mRNA expression and gene copy number loss for both *GFI1* and *TNFRSF11A* gene. These results suggest that copy number loss may explain the reduced expression of these genes seen in some TCGA tumours. Reduced CNA status may be driving reduced expression within our study, especially for *TNFRSF11A* which was selected based on this premise. Future application of DNA targeted *in situ* technologies will complement RNAscope^®^ for profiling gene copy number and expression at the single cell level.

Assessment of intra-tumoural heterogeneity within our study was achieved by combining an image analysis tool with RNAscope^®^ to provide a standardised, objective method to quantify mRNA expression levels (mRNA signals/cell) for *GFI1* and *TNFRSF11A*. Our data showed *GFI1* and *TNFRSF11A* were expressed at a significantly higher level in carcinoma cells compared to non-carcinoma cells (lymphocytes, stromal cells and normal cells). Thus, by analysing tumours at the cellular level we were able to demonstrate that reduced mRNA expression levels measured in patients with poorer prognosis were specifically due to carcinoma cells within the tumour and not due to contamination of tumour samples with non-carcinoma cells. It is therefore possible that reduced expression of these genes in patients with poor prognosis found by the TCGA study [7] was due in-part to over-representation of non-carcinoma cells in the analysed samples.

Limitations with using RNA *in situ* hybridisation include potential issues surrounding RNA recovery and degradation in FFPE material, which have been well documented and encompass pre-analytical variables of specimen fixation, processing and storage prior to TMA construction [32–34]. These factors are known to influence the quality and abundance of RNA within FFPE samples [33]. However, the RNAscope^®^ probes are specifically designed to overcome these issues [15].

In our study, archival FFPE material ranging between 10–15 years old was used. Despite the age of FFPE material, quality mRNA was present in the majority of the TMA cores. However, negative or low expression for positive control mRNA did exist in a minority of cases indicating sub-quality, lowly abundant RNA. No information regarding pre-analytical variables (e.g. tissue handling; tissue fixation times; tissue processing procedures) was available that could help account for these observations.

In conclusion, this study shows the advantages of an mRNA *in situ* hybridisation technique (RNAscope^®^) for validating potential gene expression biomarkers from published studies, circumventing the potential limitations associated with qPCR and immunohistochemistry. This study demonstrates RNAscope^®^ as a promising method to visualise and quantify mRNA expression of candidate biomarkers on archival FFPE colorectal cancer cases. This will allow researchers to carry-out retrospective studies on archival FFPE material, which have a wealth of pathological, clinical and follow-up information available, to investigate and validate candidate biomarkers. Our selection of candidate biomarkers using copy number changes provides a level of standardisation that can be implemented across different patient cohorts when investigating biomarkers to overcome the impacts of tumour heterogeneity in gene expression studies. This study found significant associations for altered mRNA expression for the genes, *GFI1* and *TNFRSF11A,* with a selection of poor prognostic features that were consistent with those identified by a large TCGA study. To our knowledge, this is the first study to assess the intercellular expression patterns of these candidate prognostic markers in colorectal tumours. Quantitative data generated by RNAscope^®^ overcame potential affects from tumour heterogeneity, permitting direct association of carcinoma specific mRNA expression level changes with clinicopathological outcomes. Future larger studies are required to confirm the link between *GFI1* and *TNFRSF11A* and patient outcome, and to determine the role of these genes in colorectal cancer development.

## MATERIALS AND METHODS

### Patient samples

A cohort of 112 primary colorectal adenocarcinoma cases of varying histology, grade, age and gender were obtained from the Christchurch Cancer Society Tissue Bank (Ethics approval #16STH92) [35]. Before obtaining tissue samples, informed consent was obtained from each patient. A detailed description of patient clinicopathological features is shown in Table 6. Two tissue microarrays (TMA) were utilised in this study. The TMAs were made from 102 cases and consisted of duplicate 1mm cores mined from FFPE tumour samples that were representative of the tumour stage at diagnosis. An additional ten cases used in the study were represented by whole histological sections from formalin fixed paraffin embedded (FFPE) tissue.

**Table 6:** Summary of the clinicopathological features of the 112 colorectal patient samples

Clinicopathological Features	Number
**Patients** (*n*)	112
**Mean age** (years ± SD)	68.97 ± 12.13
**Gender**	
Male	69
Female	41
ND	2
**Histology**	
Adenoma	10
Adenocarcinoma	99
Carcinoma	1
ND	2
**Mean tumour size, mm ± SD**	45.31 ± 16.00
**Metastasis Present**	
Yes	35
No	72
ND	5
**Site**	
Left (Distal)	61
Right (Proximal)	49
ND	2
**Primary Site**	
Ascending	42
Caecum	5
Transverse	1
Descending	12
Sigmoid	21
Rectum	28
ND	3
**Tumour Grade**	
1	5
2	72
3	17
Adenoma	10
ND	8
**AJCC Pathologic Tumour Stage**	
T0	9
T1	5
T2	22
T3	64
T4	9
ND	3
**AJCC Nodal Stage**	
N0	72
N1	21
N2	16
ND	1
**AJCC Metastatic Stage**	
M0	2
M1	5
MX	98
ND	5
**AJCC Tumour Stage Group**	
0	9
1	21
2	42
3	33
4	4
ND	3

Abbreviations: n, Number of; SD, standard deviation; ND, No Data; AJCC, American Joint Committee on Cancer

### Identification of prognostic gene markers using published TCGA data

The Cancer Genome Atlas (TCGA) Network published a comprehensive study of gene expression and genomic changes in a series of colon and rectal tumours [12]. This analysis revealed the expression of 1313 genes associated with prognostic features, such as tumour stage, lymphatic invasion, metastasis, lymph node involvement, and histology. We selected two genes, *GFI1* and *TNFRSF11A,* from this study that were highly ranked (1st and 12th, respectively) among the genes associated with tumour aggressiveness, respectively (see Table 7).

**Table 7:** *GFI1* and *TNFRSF11A* mRNA expression and associations with clinicopathological features from colorectal cancer cases in the TCGA

Gene	GFI1	TNFRSF11A
Tumour Aggressiveness Direction	**−**1	**−**1
Compound *p*-value	1.00E-16	8.18E-11
Lymphatic Invasion	0.0475	0.149
Histological Type	1.91E-10	1.06E-05
Vascular Invasion	0.207	0.593
Fraction Positive Lymph Nodes	1.54E-05	2.12E-06
Tumor Stage	6.96E-07	1.27E-05
Distant Metastasis	3.86E-06	6.03E-04

*GFI1* is a transcription repressor previously associated with intestinal epithelial cell differentiation [36]. *TNFRSF11A* is a member of the TNF receptor super family, and plays a major role in bone remodelling and immunity [30]. In addition, *TNFRSF11A* was selected based on the observation that both reduced copy number (18q deletion) and reduced expression were associated with aggressive tumours. Candidate prognostic gene markers showing a correlation between copy number alterations (CNA) and expression are more likely to be evident across a significant proportion of the tumour, and thus circumvent potential non-reproducibility associated with tumour heterogeneity [8]. Therefore, our hypothesis was that detection of CNA, especially DNA copy loss, is less likely if only present in a small proportion of tumour cells.

### Public array datasets

Expression data, DNA copy number data and clinicopathological data for 218 colorectal adenocarcinoma patients was downloaded from The Cancer Genome Atlas (TCGA) network (https://cancergenome.nih.gov/; Download: 10 August 2016) and analysed. We also downloaded and compiled microarray expression data for *GFI1* and *TNFRSF11A*, and putative copy number calls for *GFI1* and *TNFRSF11A,* using the cBioPortal for Cancer Genomics (http://www.cbioportal.org; Download: 10 August 2016) [37, 38]. Tests for association with clinicopathological parameters were carried out using the statistical programme R [39].

### RNA *in-situ* hybridisation (RNAscope^®^)

The mRNA expression levels for *GFI1, TNFRSF11A* and *MLH1* were investigated using the RNAscope^®^ 2.0 Assay (Advanced Cell Diagnostics, Inc. (Hayward, CA) [15]. The probes for *GFI1, TNFRSF11A* and *MLH1* were selected from the ACD RNAscope^®^ probe catalogue for human species. The assay was optimized and performed according to manufacturer's instructions. Briefly, for both individual tissue and TMA specimens, sections were cut at 4μm thickness and placed on SuperFrost Plus glass slides, before deparaffinising in a series of xylene and 100% ethanol steps. Each section was then subjected to a series of pre-treatment steps before progressing onto hybridisation with target probes for their respective gene. Hybridisation involved placing 4–6 drops from a Ready-To-Use bottle (approximately 150 μL) of the individual target probes onto the slide, enough to cover tissue sections. Positive (*PPIB*) and negative (DAB) control probes were hybridised to additional sections. Slides were then covered in a HybEZ™ Humidity Control Tray and placed in the HybEZ™ Oven and incubated at 40° C for 2 hours. After this time, a horseradish peroxidase-based signal amplification system was applied to consecutively hybridise pre-amplifier and several amplifiers to the target probes before colour development using diaminobenzedine (DAB). Slides were then counterstained with Gill's Haematoxylin, dehydrated and cleared before being mounted, coverslipped and assessed microscopically. Slides were determined to be positive for mRNA expression if brown punctuate dots could be seen within cells.

### Immunohistochemistry for MLH1

Immunohistochemistry was performed using the Roche VENTANA BenchMark ULTRA System (Ventana Medical Systems, Inc, Tucson, Arizona, USA). For both individual tissue and TMA specimens, 4 μm sections were cut, placed onto SuperFrost Plus glass slides, deparaffinised in xylene and placed in 100% ethanol. Slides were loaded onto the BenchMark ULTRA System and subjected to heat induced epitope retrieval using “Cell Conditioner 1” at 95° C for 64 minutes, followed by incubation with one drop of the VENTANA pre-dilute MLH1 (M1) mouse monoclonal primary antibody (final concentration 1.4 μg/mL) for 16 minutes at 36° C. Detection was achieved with the ultraView Universal DAB Detection Kit, before each section was counterstained with haematoxylin, coverslipped, and assessed microscopically.

### Image acquisition

Images were captured using a Zeiss Apotome Microscope and associated software (AxioVersion 4.5. Apotome software, Carl Zeiss Microscopy, LLC, Thornwood, New York, USA). Three representative images of carcinoma cells, normal epithelial cells, lymphocytes and stromal fibroblasts were captured for each tumour case for each individual probe at 40x resolution.

### Microscopic assessment of immunohistochemistry and RNAscope^®^ assays

The mRNA expression levels for all three probes were visually assessed by a cytologist (AM-B) and a pathologist (MRW), blinded to clinicopathological data. Slides exhibiting positive mRNA expression were semi-quantitatively and manually assessed for the number of probe signals per cell for each case using the manufacturers scoring system [15]. The scores ranged between 0–4 (0 = Negative; 1 = 1–3 probes per cell at 40x magnification; 2 = 4–6 probes per cell at 40× magnification; 3 = >10 probes per cell at 40x magnification; 4 = >10 probes per cell occupying >10% of slide or at 20x magnification). An RNAscope^®^ score of >2 for the positive control gene (*PPIB*) is indicative of abundant, good quality RNA. Assessment and scoring of *MLH1* immunohistochemistry staining in specimens was carried out by a pathologist (MRW). Positive staining for *MLH1* was considered when carcinoma cell nuclei displayed any traces of brown positive staining. A negative staining was considered when carcinoma cell nuclei were negative while other types of cell nuclei were positive. ImageJ software [40] was utilised for quantifying hybridisation signals in different tumour cell types across the whole tissue sections from 10 cases. For each case, 3 × 100 cells per cell type (carcinoma, normal epithelial, lymphocytes and stromal fibroblasts) were counted. Therefore, approximately 3000 cells per cell type were counted for the 10 cases for each gene.

### Statistical analysis

The two-sample *t-*test with Satterthwaite's adjustment for unequal variances was used to test for differences in means between gene copy number and mRNA mean expression levels. Fisher's exact test was used to test homogeneity between RNAscope^®^ and immunohistochemistry. Logistic regression was used to test for associations between semi quantitative gene expression levels and clinicopathological features for RNAscope^®^ data. All tests were two sided with a *p*-value of ≤ 0.05 considered to be significant. ANOVA with Tukey post-hoc *t*-tests was used to investigate differences in mean mRNA expression levels and cell types. Analysis was performed in R 3.3.1 (Vienna, Austria).

## SUPPLEMENTARY MATERIALS FIGURE



## Abbreviations

TCGA: The Cancer Genome Atlas Network
TMA: tissue microarrays
FFPE: formalin fixed paraffin embedded
CNA: copy number alterations
AJCC: American Joint Committee on Cancer
