# Supplementary material for: Assessment of intra-tumoural colorectal cancer prognostic biomarkers using RNA in situ hybridisation
Source: Oncotarget. 2019 Feb 15;10(14):1425–39. doi: 10.18632/oncotarget.26675 (PMC6402718; doi:10.18632/oncotarget.26675)
Supplement: Supplementary file 1 [file oncotarget-10-1425-s001.pdf]

Assessment of intra-tumoural colorectal cancer prognostic biomarkers using RNA *in situ* hybridisation

SUPPLEMENTARY MATERIALS

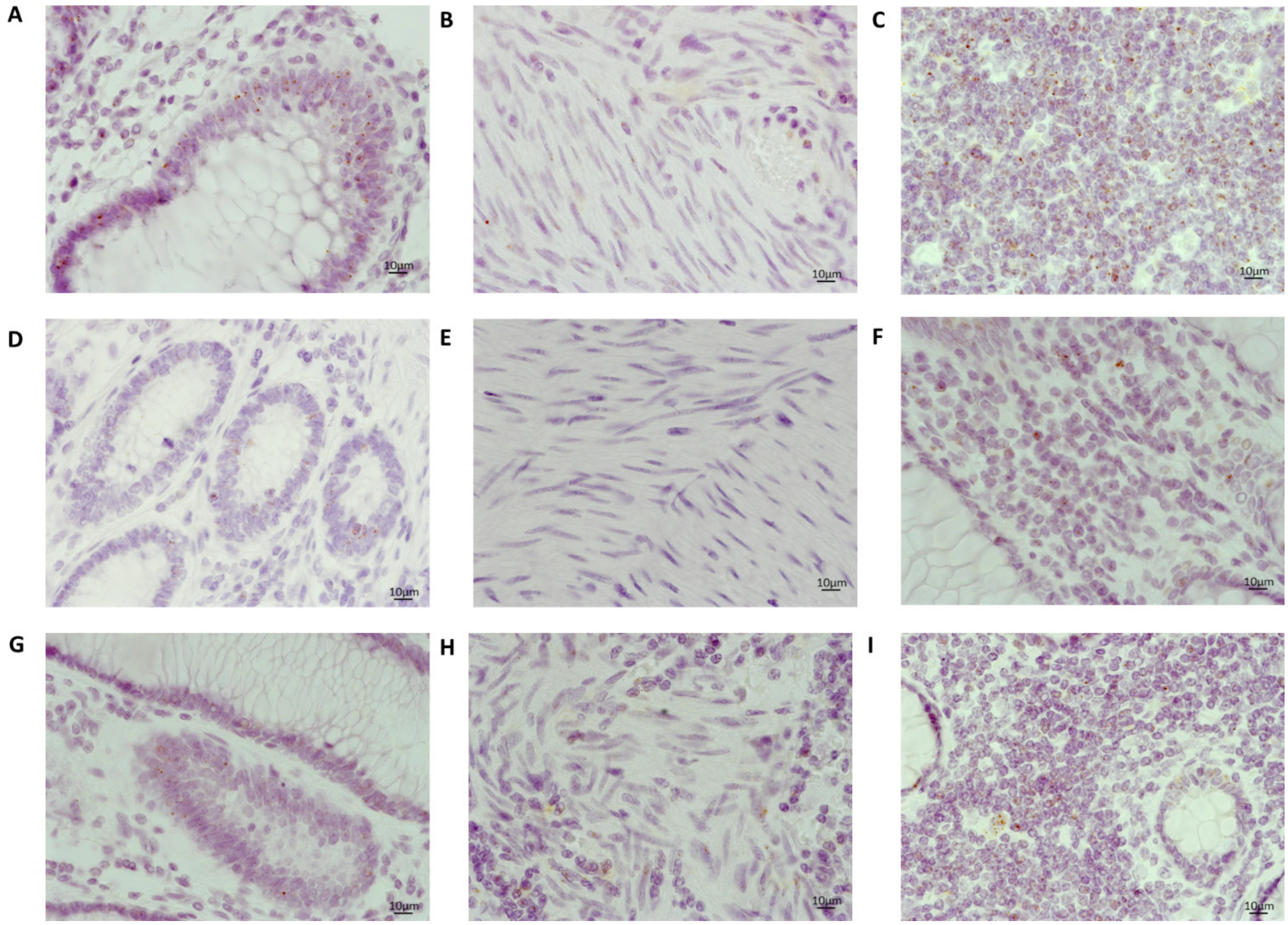

Supplementary Figure 1: Representative images of mRNA expression using RNAscope® on FFPE whole tissue section in different cell types from colorectal cancer cases at 40× magnification (**A**) *MLH1* in normal epithelial cells (**B**) *MLH1* in stromal cells (**C**) *MLH1* in lymphoid cells (**D**) *GFI1* in normal epithelial cells (**E**) *GFI1* in stromal cells (**F**) *GFI1* in lymphoid epithelial cells (**G**) *TNFRSF11A* in normal epithelial cells (**H**) *TNFRSF11A* in stromal cells (**I**) *TNFRSF11A* in lymphoid cells.
